# Alternating Hemispheric Episodes in Bilateral Rasmussen Encephalitis: The Crucial Role of Sequential Multimodal Neuroimaging

**DOI:** 10.7759/cureus.111916

**Published:** 2026-07-01

**Authors:** Amal Akammar, Hajar Ouazzani, Ismail Chaouache, Nizar EL Bouardi, Meriem Haloua, Badreeddine Alami, Youssef Alaoui Lamrani, Mustapha Maaroufi, Meryem Boubbou

**Affiliations:** 1 Mother and Child Radiology, University Hospital Center Hassan II, Sidi Mohamed Ben Abdellah University, Fes, MAR; 2 Central Radiology, University Hospital Center Hassan II, Sidi Mohamed Ben Abdellah University, Fes, MAR; 3 Radiology, University Hospital Center Hassan II, Fes, MAR

**Keywords:** bilateral hemispheric, encephalitis, epilepsy, rasmussen encephalitis, serial mri

## Abstract

Rasmussen encephalitis (RE) is a rare, progressive inflammatory encephalopathy typically characterized by drug-resistant focal epilepsy, cognitive decline, and unihemispheric brain atrophy. Bilateral involvement remains an exceptional clinical entity with limited documentation in medical literature. We present the case of a 2.5-year-old female who exhibited two separate episodes of febrile status epilepticus impacting alternating hemispheres over a six-month period. Serial neuroimaging was pivotal in tracking the disease; the initial MRI revealed right-sided cytotoxic edema and cortical thickening, while a subsequent scan unveiled new contralateral left-sided cortico-subcortical signal alterations alongside progressive right hemispheric atrophy. Continuous bilateral epileptic activity was further corroborated by electroencephalography (EEG). This case highlights that, while RE is classically unilateral, clinicians must maintain a high index of suspicion for bilateral variants, where sequential multimodal neuroimaging proves indispensable for timely diagnosis, prognostic assessment, and therapeutic planning.

## Introduction

Rasmussen encephalitis (RE) is a rare, progressive inflammatory encephalopathy that predominantly affects pediatric patients. It is classically characterized by pharmacoresistant focal epilepsy, progressive hemiparesis, and cognitive decline, driven by typically unihemispheric brain atrophy [1,2]. The clinical course typically unfolds in three phases: a prodromal stage with infrequent seizures, an acute stage marked by frequent seizures, epilepsia partialis continua, and progressive neurological deterioration, followed by a chronic residual stage with stable neurological deficits [3]. Diagnosis relies on a multimodal framework integrating clinical, electroencephalography (EEG), and neuroimaging parameters, as formalized in the European consensus criteria [4]. Serial MRI is indispensable for monitoring RE progression; it typically tracks sequential cortical volume loss over time, T2/fluid-attenuated inversion recovery (FLAIR) signal alterations, and focal atrophy, yet the early inflammatory stage can easily masquerade as herpetic encephalitis, stroke, or other autoimmune conditions [5,6]. Bilateral RE, whether presenting simultaneously or sequentially, is an exceptional variant with fewer than 20 cases documented worldwide [7,8]. Detecting bilateral involvement is clinically profound, as it fundamentally alters standard surgical paradigms, often precluding traditional resective hemispheric surgery and necessitating an individualized, multidisciplinary assessment alongside aggressive immunomodulatory therapies [4].

The primary objective of this report is to delineate the sequential, cross-hemispheric neuroimaging features and diagnostic milestones of this rare bilateral variant of RE, thereby highlighting the critical challenges in clinical decision-making and therapeutic reorientation.

## Case presentation

A 2.5-year-old female with an unremarkable personal and family medical history and age-appropriate psychomotor development (head control at four months, independent walking at 14 months) was initially admitted at age two following an episode of febrile status epilepticus (SE). The event presented as prolonged left hemicorporeal clonic seizures lasting over an hour during a high fever (38.6°C).

On admission, generalized tonic-clonic activity was controlled via rectal diazepam and a continuous midazolam infusion. Post-ictal evaluation revealed altered consciousness (Glasgow Coma Scale (GCS) of 9) and a transient left monoplegia. Initial laboratory workup showed a marked increase, with a white blood cell count of 31,200/µL (normal range: 4,000-11,000/µL), accompanied by a normal metabolic panel, including serum electrolytes, renal function, and liver enzymes (Table 1). An urgent unenhanced brain CT performed on admission was strictly normal.

**Table 1:** Laboratory findings during the two hospital admissions

Parameter	First Admission	Second Admission	Interpretation
WBC (White Blood Cells)	31,200/µL (normal: 4,000-11,000/µL)	5,000/µL (normal: 4,000-11,000/µL)	Marked leukocytosis, suggesting an acute inflammatory/infectious response
CRP (C-Reactive Protein)	6 mg/L (normal: <5 mg/L)	188 mg/L (normal: <5 mg/L)	Markedly elevated, consistent with a severe systemic inflammatory response
Procalcitonin	Not available	38 ng/mL (normal: <0.5 ng/mL)	Markedly elevated, initially suggesting bacterial infection
Capillary Blood Glucose	0.9 g/L (normal: 0.7-1.1 g/L)	0.2 g/L (normal: 0.7-1.1 g/L)	Severe hypoglycemia, immediately corrected with IV glucose

A lumbar puncture (LP) yielded clear cerebrospinal fluid (CSF) with nine white cells/µL (normal range: <5 cells/µL), 80 red cells/µL (normal range: 0 cells/µL), and a negative FilmArray meningitis/encephalitis panel (which explicitly included negative PCR results for HSV-1 and HSV-2). This mild pleocytosis was interpreted as a non-specific finding, secondary to a minor traumatic tap or reflective of the underlying neuroinflammatory process. While a repeat LP was not performed, infectious causes were definitively excluded based on the negative initial panel, broader serological screenings, and the subsequent clinical and neuroimaging evolution.

Empiric therapy with ceftriaxone and acyclovir was initiated. Due to breakthrough left-sided seizures on Day 3, valproate was added. A repeat LP on Day 6 remained unremarkable, and an EEG showed mild right hemispheric slowing without periodic lateralized epileptiform discharges (PLEDs) or temporal epileptiform abnormalities typically associated with herpes simplex encephalitis.

A brain MRI obtained on Day 7 of the first hospitalization revealed a profound loss of grey-white matter differentiation throughout the right hemisphere, characterized by T2/fluid-attenuated inversion recovery (FLAIR) hyperintensity and diffusion restriction indicative of cytotoxic edema. This was accompanied by diffuse right cortical thickening, right thalamic FLAIR hyperintensity, and showed no pathological contrast enhancement (Figure 1).

**Figure 1:**
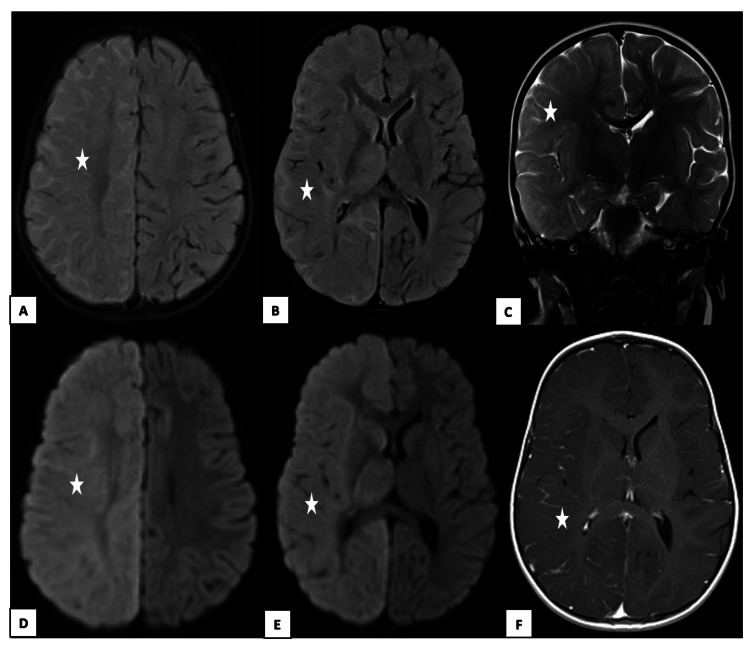
Brain MRI findings in the acute inflammatory phase of Rasmussen encephalitis Axial fluid-attenuated inversion recovery (FLAIR) images (A, B) demonstrate right hemispheric cortico-subcortical hyperintensities (★), not confined to any vascular territory, with diffuse cortical thickening, loss of the gray-white matter interface, and ipsilateral thalamic FLAIR hyperintensity. Coronal T2-weighted image (C) confirms diffuse right hemispheric cortical thickening (★) with loss of gray-white matter differentiation, consistent with cytotoxic edema in the acute inflammatory phase. Axial diffusion-weighted images (D, E) demonstrate diffusion restriction within the right hemispheric cortico-subcortical lesions (★). Axial T1-weighted post-contrast image (F) shows the absence of pathological enhancement within the right hemispheric signal abnormalities (★).

With infectious causes effectively excluded by the negative CSF assays, the diagnosis shifted toward either severe SE-induced brain injury or an early presentation of Rasmussen encephalitis. Clinically, the progressive nature of the neurological deficits and subsequent neuroimaging showing progressive unilateral cortical atrophy strongly favored early RE over postictal injury. The patient completed a 21-day course of acyclovir for presumed herpetic meningoencephalitis. She was discharged with residual left hemiparesis and facial palsy, managed with a maintenance regimen of valproate (23 mg/kg/day; 150 mg twice daily) and regular motor physiotherapy. Given the diagnostic uncertainty at this stage, immunotherapy was not initiated, and management focused on aggressive antiseizure therapy while awaiting definitive clinical and radiological confirmation.

Six months later, the patient was readmitted at age three following a new episode of febrile SE, which manifested atypically as right hemicorporeal focal impaired-awareness seizures. Acute severe hypoglycemia with a capillary blood glucose of 0.2 g/L (normal range: 0.7-1.1 g/L) was identified on arrival and immediately corrected with intravenous glucose. Although severe hypoglycemia can cause encephalopathy, hypoglycemic brain injury was excluded as a primary cause of the subsequent deficits due to the immediate metabolic correction, the absence of typical bilateral neuroimaging changes, and the subsequent clinical evolution marked by progressive, unilateral cortical atrophy highly specific to RE.

The post-ictal examination showed a GCS of 9, right monoplegia, and neck stiffness. Due to worsening respiratory function and heavy bronchial congestion, she was transferred to the Pediatric Intensive Care Unit (PICU), where a respiratory FilmArray-isolated human rhinovirus and respiratory syncytial virus.

Refractory myoclonic seizures emerged on Day 4, requiring the addition of phenobarbital to her valproate and midazolam regimen. Markedly elevated inflammatory markers, with a C-reactive protein (CRP) peaking at 188 mg/L (normal range: <5 mg/L) and procalcitonin at 38 ng/mL (normal range: <0.5 ng/mL), prompted an escalation of antibiotics to imipenem-cilastatin and ciprofloxacin.

An urgent brain CT performed on Day 1 of this second admission demonstrated prominent right cerebral atrophy with associated hypodense sequelae from the initial hemispheric insult, raising the suspicion of Rasmussen encephalitis (Figure 2).

**Figure 2:**
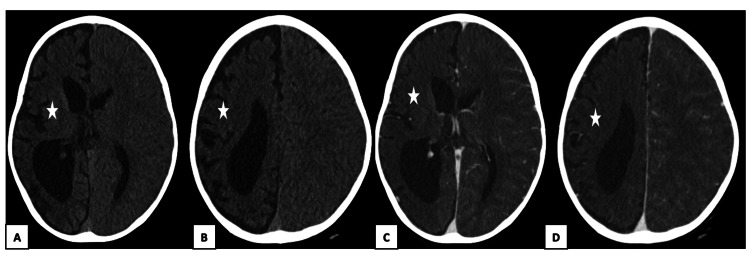
Brain CT scan Axial non-contrast CT images (A, B) demonstrate a right hemispheric cortico-subcortical hypodense area (★), not confined to any vascular territory, associated with widening of the right cortical sulci and ex-vacuo dilatation of the right lateral ventricle, reflecting parenchymal volume loss in the adjacent cerebral hemisphere. Axial post-contrast CT images (C, D) confirm the absence of pathological enhancement within the right hemispheric hypodense lesions (★), consistent with the non-enhancing pattern characteristically observed in Rasmussen encephalitis.

The follow-up brain MRI demonstrated the new onset of a diffuse T2 and FLAIR hyperintensity encompassing the left cerebral hemisphere, associated with prominent cortical thickening and significant diffusion restriction, without any abnormal contrast enhancement following intravenous gadolinium administration. This acute involvement extended to the left thalamus and hippocampus, while the posterior fossa structures were completely spared. Concurrently, it confirmed marked right cerebral atrophy with widening of the ipsilateral sulci, representing the chronic sequelae of the initial hemispheric insult (Figure 3).

**Figure 3:**
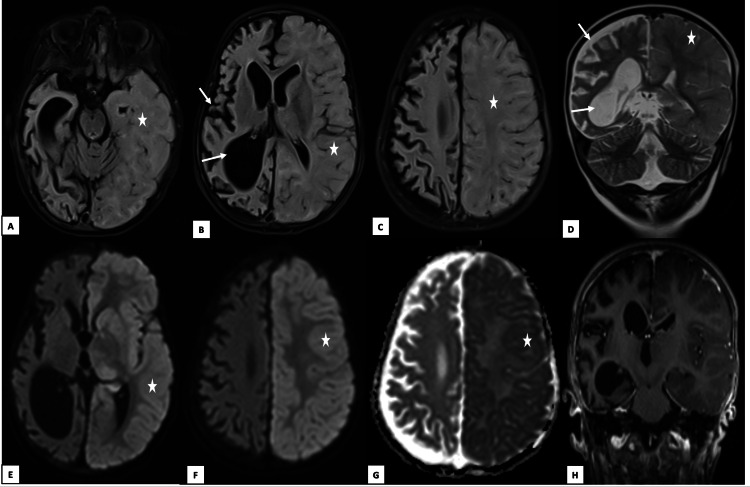
Follow-up brain MRI findings during the second admission (A, B, C) Axial fluid-attenuated inversion recovery (FLAIR) images demonstrate extensive left hemispheric cortico-subcortical hyperintensities (★), not confined to any vascular territory, with diffuse cortical thickening and ipsilateral pulvinar involvement (arrows in B). (D) Coronal T2-weighted image shows diffuse left hemispheric cortical thickening (★) and contralateral right sulcal widening with passive dilatation of the right lateral ventricle (arrows), reflecting chronic right hemispheric volume loss without signal abnormality. (E, F) Axial diffusion-weighted images (DWI) demonstrate diffusion restriction within the left hemispheric cortico-subcortical lesions (★), reflecting excitotoxic cellular swelling and early inflammatory necrosis. (G) Axial apparent diffusion coefficient (ADC) map confirms true diffusion restriction within the left hemispheric lesions (★), with corresponding signal reduction. (H) Coronal T1-weighted post-contrast image shows the absence of pathological enhancement within the left hemispheric signal abnormalities.

An EEG demonstrated a poorly organized and slowed waking background activity with a pronounced asymmetry, characterized by right-sided theta rhythms. No paroxysmal abnormalities, spike-and-wave complexes, or electroclinical seizures were recorded during the examination (Figure 4). 

**Figure 4:**
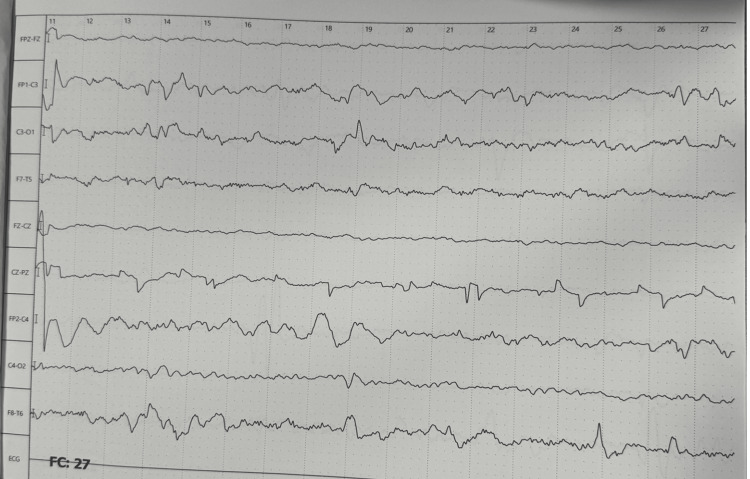
Electroencephalogram (EEG) Waking electroencephalogram (EEG) demonstrating significant background asymmetry. The montage shows poorly organized, slowed background activity characterized by asymmetric right-sided theta rhythms, without paroxysmal abnormalities or ictal discharges.

Given the striking radiological evolution alongside the clinical progression of drug-resistant focal seizures affecting alternating hemispheres, the diagnosis of bilateral Rasmussen encephalitis was considered most consistent with the patient's presentation. The demonstration of an active inflammatory phase in the left hemisphere, emerging sequentially after structural damage and subsequent atrophy had already anchored in the right hemisphere, The demonstration of an active inflammatory phase in the left hemisphere, emerging sequentially after structural damage and subsequent atrophy had already anchored in the right hemisphere, provided the pathognomonic evidence required to confirm this rare bilateral entity. While the hemi-hemiconvulsion-hemiplegia-epilepsy (HHE) syndrome was initially discussed due to the presentation of the first episode, the cross-hemispheric progression captured on serial neuroimaging strongly argued against a simple unilateral vascular process. Although postictal or status-related injury remains an important differential diagnosis that can mimic these changes, the prolonged longitudinal evolution and progressive contralateral involvement heavily favored a progressive bilateral encephalopathy, shifting the therapeutic strategy toward aggressive medical management.

By Day 23, the patient was conscious but exhibited a severe regression in communication skills with an absence of verbal responsiveness, alongside a fixed leftward head and gaze deviation, severe axial hypotonia, and an inability to maintain an upright posture. Neurological examination noted relative hypotonia in the left upper limb, bilateral lower limb hypertonia with resistance to passive movement, and bilateral equivocal Babinski sign.

Antiepileptic therapy was optimized by adding levetiracetam, and the patient was discharged on a triple-drug regimen of clobazam, valproate, and levetiracetam with close outpatient neuro-pediatric follow-up, which successfully achieved complete seizure control prior to discharge. Due to the very recent nature of this presentation, specialized immunomodulatory therapies, such as corticosteroids, intravenous immunoglobulin (IVIG), plasmapheresis, rituximab, or tacrolimus, were not initiated during the acute hospitalization phase. However, these therapies remain planned and are currently being evaluated as part of the patient's upcoming outpatient specialized neuro-pediatric management strategy.

## Discussion

RE is a progressive focal neuroinflammatory disorder driven by a T-cell-mediated autoimmune cascade, where cytotoxic CD8+ T lymphocytes target neurons and astrocytes [1,2]. This process leads to irreversible hemispheric destruction, resulting in pharmacoresistant focal epilepsy, epilepsia partialis continua, progressive hemiparesis, and cognitive decline [3]. Establishing the diagnosis requires a multimodal approach combining clinical, electroencephalographic, and neuroimaging findings, according to the European consensus criteria [4].

The clinical course of our patient highlights the challenges of the hyperacute, early inflammatory phase of RE, where radiological features contrast sharply with the classic late atrophic stage. Bien et al. established a unidirectional, four-stage sequential MRI classification correlated with histopathology: Stage 1 involves cortical swelling with T2/FLAIR hyperintensity (acute inflammatory edema); Stage 2 demonstrates persistent hyperintensity with volume normalization; Stage 3 shows progressive atrophy with sustained hyperintensity; and Stage 4 leads to advanced hemispheric atrophy with signal normalization, signaling burned-out gliosis [5]. Quantitative analysis shows that T lymphocyte and reactive astrocyte densities peak during these earliest stages and decline as tissue loss progresses [5]. Because different cortical regions undergo this process asynchronously, a single MRI scan typically reveals heterogeneous hemispheric involvement [5]. This temporal profile underscores why immunomodulatory therapies are most effective within the first 24 months of disease onset, targeting active inflammation before widespread neuronal death occurs [5].

At initial presentation, the patient's right hemispheric cytotoxic edema, T2/FLAIR hyperintensity, diffusion restriction, and cortical thickening matched Stage 1 features. These findings frequently mimic post-ictal changes from prolonged focal status epilepticus, herpetic meningoencephalitis, or acute hemispheric ischemic stroke [7,8]. The observed diffusion restriction likely stems from a dual mechanism: acute excitotoxic cellular swelling induced by status epilepticus and early inflammatory necrosis from the encephalitic process. Distinguishing early RE from other acute hemispheric lesions relies heavily on topography. The predominant fronto-insular distribution of signal abnormalities is a well-documented marker of RE, differing from the temporo-mesial tropism of herpes simplex encephalitis and the strict vascular territories of ischemic strokes [6,7]. In RE, these signal alterations typically originate focally within the frontal or temporal lobes before spreading across the affected hemisphere, a pattern described as a "march across the hemisphere" while characteristically sparing the contralateral side during the initial years [5,6].

Managing the initial presentation as presumed herpetic meningoencephalitis represents a frequent diagnostic pitfall [1,3]. In this case, persistently negative CSF PCR assays for herpes simplex virus and the absence of typical temporal lobe involvement excluded an infectious etiology. Furthermore, herpetic encephalitis typically displays more pronounced diffusion restriction within the lesions compared to RE [6]. On MRI, RE is characterized by T2/FLAIR cortical and subcortical hyperintensities in fronto-insular and perisylvian regions, progressive unilateral atrophy, and a general lack of gadolinium enhancement, which occurs only transiently during acute exacerbations in adult-onset forms [5,6]. Complementary functional investigations can aid diagnosis: SPECT (Tc-99m HMPAO) and FDG-PET can detect hemispheric hypoperfusion and hypometabolism before structural changes appear on MRI, occasionally demonstrating crossed cerebellar diaschisis [6]. Additionally, MR spectroscopy typically reveals reduced NAA and choline levels alongside elevated myoinositol and glutamate/glutamine ratios, reflecting neuronal depletion and microglial activation [6].

The atypical dimension of this case lies in the sequential, contralateral hemispheric involvement detected on neuroimaging six months later. While the right hemisphere had progressed to chronic atrophy, the follow-up MRI revealed a new wave of left cortico-subcortical FLAIR hyperintensities and cortical thickening, characteristic of contralateral Stage 1-2 changes. This sequential, bilateral involvement defines bilateral RE, a rare variant documented in a few pediatric cases worldwide [4,5]. The underlying pathophysiology remains unclear; current hypotheses suggest widespread, systemic autoimmune activation with asynchronous regional onset, transcallosal propagation of inflammation, or independent bilateral autoimmune triggers [9,10]. Serial MRI proved essential to detect this asynchronous progression, confirming that regular neuroimaging is required in RE to screen for delayed contralateral extensions that alter both prognosis and management [5,6].

The patient's EEG corroborated this bilateral architecture, demonstrating a marked background asymmetry and slowed waking activity characterized by right-sided theta rhythms, without any paroxysmal abnormalities. Documenting a bilateral epileptic signature alters the therapeutic strategy. While hemispherotomy is the gold-standard treatment for unihemispheric RE, offering definitive seizure control in selected candidates [9], it is strictly contraindicated in bilateral disease due to the severe motor and cognitive deficits it would induce [3,4]. Management must rely instead on aggressive immunomodulation, combining high-dose corticosteroids, IVIG, and plasmapheresis, or long-term immunosuppression with rituximab or tacrolimus, despite limited data for bilateral variants [4,5].

The HHE syndrome, considered in the differential diagnosis, shares phenotypic features with this presentation. HHE typically affects children under four years, presenting with prolonged febrile hemicorporeal convulsions followed by flaccid hemiplegia and chronic epilepsy [10]. A pathophysiological link between HHE and early RE has been suggested; the intense inflammatory response triggered by prolonged febrile status epilepticus might unmask or accelerate RE in an immunologically vulnerable host [1,10].

## Conclusions

The early inflammatory phase of RE frequently mimics other acute hemispheric disorders. Serial neuroimaging, tracking the Bien stages and potential cross-hemispheric involvement, remains the standard for monitoring disease evolution. Recognizing sequential bilateral involvement is of paramount practical importance, as it precludes contraindicated localized surgical resections and mandates a shift toward systemic medical management. In our patient, this timely recognition led to a successful triple anti-seizure regimen, achieving complete seizure control at discharge, with systemic immunomodulatory therapy planned for the subsequent follow-up.
